# Case Report: Novel Monitoring for Anaerobic Conditions Detected by Respiratory Quotient in a Critically Ill Pediatric Patient

**DOI:** 10.3389/fped.2022.874969

**Published:** 2022-04-07

**Authors:** Kenichiro Hayashi, Hikoro Matsui

**Affiliations:** Department of Pediatrics, The University of Tokyo Hospital, Bunkyo City, Japan

**Keywords:** anaerobic metabolism, indirect calorimetry, respiratory quotient, VCO_2_, VO_2_

## Abstract

**Background:**

Hyperlactemia after cardiopulmonary bypass is associated with adverse events during the early postoperative period in children. Serum lactate levels, a standard marker of anaerobic metabolism, are determined by the production, conversion and clearance of lactate, and may lag behind the anaerobic response. Here, we report a neonatal case under anaerobic conditions after cardiac surgery, whose expired gas parameters dramatically changed before a rise in blood lactate.

**Case Presentation:**

A 23-day-old girl with tetralogy of Fallot was admitted to the pediatric intensive care unit after modified Blalock-Taussig shunt operation. As hemoconcentration increased and pleural fluid and ascites accumulated, we performed partial exchange transfusion to prevent shunt occlusion. Ten minutes after partial exchange transfusion, oxygen uptake and carbon dioxide production measured by indirect calorimetry suddenly dropped, while the respiratory quotient began to rise steeply before hyperlactatemia developed a few hours later.

**Conclusion:**

Analysis of expired gas in critically ill children can detect the transition from aerobic to anaerobic conditions before hyperlactatemia.

## Introduction

Aerobic metabolism maintains the balance between systemic oxygen (O_2_) delivery and consumption and is a key component in the management of critically ill patients. Anaerobic metabolism may arise through a mismatch between O_2_ demand and supply, a phenomenon that is often observed in pediatric patients after cardiopulmonary bypass (CPB) and can lead to hyperlactatemia, which is associated with adverse events during the early postoperative period (1–3). Serum lactate levels, a standard marker of anaerobic metabolism, generally reflect not only the results of anaerobic glycolysis, but also the conversion of lactate into pyruvate or glucose in the liver and its clearance in the liver and kidney (4). Therefore, direct analysis of O_2_ metabolism behind lactate production would provide further important information to elucidate the pathophysiology of anaerobic conditions.

Continuous and non-invasive analysis of expired gas through indirect calorimetry (IC) has been used to detect anaerobic metabolism, especially during exercise (5). Specifically, with increasing workload, IC indicates the rise of both O_2_ uptake (VO_2_) and carbon dioxide (CO_2_) production (VCO_2_), especially VCO_2_-dominant under anaerobic condition, leading to the elevation of the respiratory quotient (RQ = VCO_2_ / VO_2_). RQ elevation is also observed in the context of tissue hypoperfusion in some animal models (6–8) and is monitored in adult patients after cardiac surgery (9). Evaluating the metabolic condition by monitoring RQ is potentially useful for achieving a better understanding of the clinical state of critically ill children (10).

Here, we report a case of neonate under anaerobic condition after cardiac surgery, whose VO_2_, VCO_2_, and RQ dramatically changed before a rise in blood lactate, indicating that IC can be utilized for hemodynamic monitoring in pediatric critical care.

## Case Presentation

The patient was a 23-day-old girl (height 47 cm, weight 3.0 kg) diagnosed with tetralogy of Fallot and suffering from repeated anoxic spells. She was admitted to the PICU and was mechanically ventilated after modified Blalock-Taussig shunt operation under CPB. At the time of admission, hemoconcentration increased, and pleural fluid and ascites accumulated and, after 6 h later, hemoglobin (Hb) and hematocrit (Hct) reached 22.1 g/dL (normal range 13.4–16.6 g/dL) and 67.5% (normal range 41–53%), respectively (17.1 g/dL and 52.2% on admission, respectively). Her hemodynamic condition was consistently stable with normal blood lactate levels (normal range below 2 mmol/L).

**FIGURE 1:**
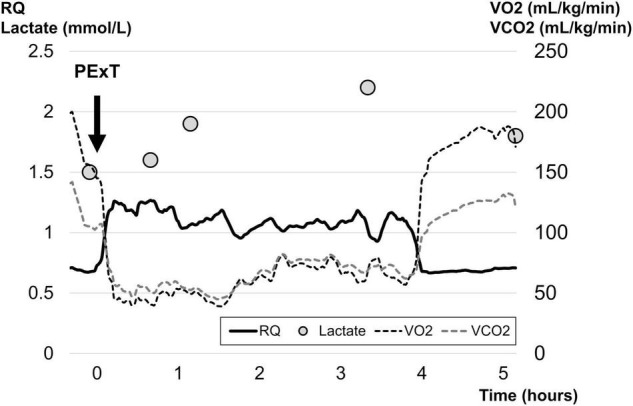
Changes in expired gas parameters and blood lactate over time. The trends of gas exchange measurements and blood lactate are presented. Ten minutes after the first PExT, both VO_2_ and VCO_2_ suddenly dropped, while RQ began to rise steeply before a rise in the level of blood lactate level up to 2.2 mmol/L a few hours after the RQ elevation. All of these parameters plateaued over the next 4 h, and then returned to the initial level. PExT, partial exchange transfusion; RQ, respiratory quotient; VCO_2_, carbon dioxide production; VO_2_, oxygen uptake.

We decided to perform partial exchange transfusion (PExT) with acetated Ringer’s solution for diluting the blood to prevent shunt occlusion due to increased blood viscosity. We performed PExT three times (25 mL/kg in total) over 2 h to minimize the influence on hemodynamics. To determine the detailed status of oxygen delivery during the procedure, we monitored VO_2_, VCO_2_, and RQ breath by breath with IC (E-COVX; GE Healthcare, Helsinki, Finland), in addition to measuring blood lactate repeatedly every 30 mins to few hours. During the transient anaerobic metabolism associated with PExT, RQ showed a very fast response compared to the change in blood lactate (Figure 1). During PExT, mild hypotension and tachycardia occurred, although cardiac function was maintained on echocardiography. Therefore, we administered minimal fluid bolus therapy to maintain organ perfusion pressure. Throughout the period, her condition was almost stable with a temperature of 36.0 ± 0.4°C and blood glucose of 150 ± 10 mg/dL without infusion of insulin.

She was extubated after her cardiorespiratory condition became stable on postoperative day 2. She was discharged from the PICU on postoperative day 5 without any complications of the surgery.

## Discussion

In the case, we recognized a novel reaction of anaerobic metabolism with a drop in VO_2_ and VCO_2_ accompanied by the steep rise of RQ right after the blood was diluted through PExT. Quantitative measuring of VO_2_, VCO_2_ and RQ is the gold standard for the assessment of energy metabolism, and increased RQ is a marker of anaerobic metabolism (11). Furthermore, the plunge of VO_2_ and VCO_2_ and the elevation of RQ have been observed under anaerobic conditions in animal models of hemodilution (7). Therefore, we firstly applied IC for evaluating the relationships between hemodilution and expired gas parameters in a neonate requiring PExT. The reaction of VO_2_, VCO_2_ and RQ occurred several hours before the mild hyperlactatemia, and then recovered as blood lactate level gradually decreased. These findings suggest that IC has a potential for a sensitive assessment of the anaerobic status in critically ill pediatric patients. On the other hand, in routine intensive care, the use of IC for energy consumption monitoring and respiratory gas analysis is still uncommon and expensive. Thus, further research is required for the practical application of IC.

Under anaerobic conditions, the drop of VCO_2_ is less pronounced than that of VO_2_, leading to the elevation of RQ (=VCO_2_ / VO_2_) (11). Even in critical states, when the systemic oxygen supply (DO_2_) is maintained at a sufficient level, both VO_2_ and the aerobic metabolite, VCO_2_, remain constant. On contrast, in the case with significant or rapid decreases of DO_2_ to a certain level, VO_2_ in peripheral tissues is proportionally limited, which indicates anaerobic metabolism (12). In our patient, the fall of VO_2_ was attributed to the impaired DO_2_ caused by hemodilution after PExT. Under anaerobic metabolism, aerobic CO_2_ production declines in parallel with the decrease in VO_2_. In the meanwhile, bicarbonates buffer protons generated anaerobically in the process of producing lactates, resulting in new anaerobic CO_2_ production, which offsets the decrease in total VCO_2_ (11). Thus, in order to detect the change from aerobic to anaerobic state, it is important to evaluate VO_2_, VCO_2_, and RQ in combination, rather than VO_2_ and VCO_2_ individually.

The early detection of the increase in RQ preceding hyperlactatemia [blood lactate value above 2 mmol/L (13)] was another noticeable finding in the patient. Under anaerobic conditions, changes in the balance between VO_2_ and VCO_2_ occur more promptly than those in the blood lactate level (11), because lactate is one of the metabolic products affected by excretion through urine and sweat or by uptake in liver (14). The rise in VO_2_ and VCO_2_ before hyperlactatemia is observed during incremental exercise (15) or in patients with inadequate perfusion during CPB (16), which means that the trend of VO_2_, VCO_2_, and RQ is potentially a more sensitive marker of anaerobic metabolism than blood lactate.

In conclusion, we report a steep rise in RQ before hyperlactatemia under anaerobic metabolism in a pediatric patient after cardiac surgery through IC monitoring. Expired gas analysis in critically ill children has the potential to detect the transition from aerobic to anaerobic conditions before hyperlactatemia occurs. This needs to be further investigated by observational studies or randomized controlled trials in the future.

## Data Availability Statement

The raw data supporting the conclusions of this article will be made available by the authors, without undue reservation.

## Ethics Statement

The studies involving human participants were reviewed and approved by the ethical committee in the University of Tokyo for publication with informed consent [Institutional Review Board No: 2701-(5)]. Written informed consent to participate in this study was provided by the participants’ legal guardian/next of kin. Written informed consent was obtained from the minor(s)’ legal guardian/next of kin for the publication of any potentially identifiable images or data included in this article.

## Author Contributions

KH contributed to the data collection and drafted the initial manuscript. HM supervised the manuscript. Both authors agree to be accountable for the content of the work and approved the submitted version.

## Conflict of Interest

The authors declare that the research was conducted in the absence of any commercial or financial relationships that could be construed as a potential conflict of interest.

## Publisher’s Note

All claims expressed in this article are solely those of the authors and do not necessarily represent those of their affiliated organizations, or those of the publisher, the editors and the reviewers. Any product that may be evaluated in this article, or claim that may be made by its manufacturer, is not guaranteed or endorsed by the publisher.
